# Efficacy of Cryoballoon Ablation for Atrial Fibrillation and Recurrence Predictors in an Asian Cohort

**DOI:** 10.3390/jpm12050732

**Published:** 2022-04-30

**Authors:** Shang-Ju Wu, Cheng-Hung Li, Chi-Jen Weng, Jiunn-Cherng Lin, Yu-Shan Chien, Yi-Huei Chen, Ching-Heng Lin, Yu-Cheng Hsieh, Jin-Long Huang, Li-Wei Lo, Yenn-Jiang Lin, Shih-Ann Chen

**Affiliations:** 1 Cardiovascular Center, Taichung Veterans General Hospital, Taichung 40705, Taiwan; alanwu0206@gmail.com (S.-J.W.); scott91695@gmail.com (C.-J.W.); horrisong@gmail.com (J.-C.L.); ruby_chien_2@yahoo.com (Y.-S.C.); golden@vghtc.gov.tw (J.-L.H.); epsachen@ms41.hinet.net (S.-A.C.); 2 Department of Internal Medicine, Faculty of Medicine, Institute of Clinical Medicine, National Yang Ming Chiao Tung University School of Medicine, Taipei 11217, Taiwan; gyrus1975@gmail.com (L.-W.L.); linyennjiang@gmail.com (Y.-J.L.); 3 Department of Data Science and Big Data Analytics, and Department of Financial Engineering, Providence University, Taichung 43301, Taiwan; 4 Department of Internal Medicine, Taichung Veterans General Hospital Chiayi Branch, Chiayi 60090, Taiwan; 5 Department of Medical Research, Taichung Veterans General Hospital, Taichung 40705, Taiwan; chenyh@vghtc.gov.tw (Y.-H.C.); epid@vghtc.gov.tw (C.-H.L.); 6 Department of Post-Baccalaureate Medicine, National Chung Hsing University School of Medicine, Taichung 40227, Taiwan; 7 Department of Medical Education, Taichung Veterans General Hospital, Taichung 40705, Taiwan; 8 Heart Rhythm Center and Division of Cardiology, Department of Medicine, Taipei Veterans General Hospital, Taipei 11217, Taiwan

**Keywords:** atrial fibrillation, cryoballoon ablation, recurrence, Asian

## Abstract

Background: Cryoballoon ablation (CBA) for atrial fibrillation (AF) is a rhythm control procedure used in clinical trials, mostly in Western countries. Its efficacy and the predictors of AF recurrence after CBA remain unclear for Asian populations. We aimed to investigate the efficacy of CBA and the predictors of AF recurrence after CBA in Asian AF patients. Methods: We included consecutive AF patients undergoing CBA for rhythm control between 2014 and 2020. The baseline characteristics, including AF types, symptom severity, and left atrial diameter (LAD), were analyzed. Holter’s monitoring and 12-lead ECG were performed to document AF recurrence. A multivariate Cox hazards regression model was used to evaluate the risk of AF recurrence. Results: A total of 120 AF patients (aged 61.9 ± 9.3 years) were included. The percentage of patients free from AF in the year following CBA was 74.2%. Among the three independent predictors of AF recurrence within one year were the presence of persistent AF (*p =* 0.025), an LAD ≥ 4.75 cm (*p* = 0.016), and pre-procedural cardioversion (*p* = 0.025). All patients survived and none had a stroke after CBA. Conclusion: CBA for AF is an effective and safe procedure in Asian populations. The presence of persistent AF, an LAD ≥ 4.75 cm, and severe symptoms are predictors of AF recurrence in the year following CBA.

## 1. Introduction

Atrial fibrillation (AF) is the most common sustained arrhythmia, resulting in both significant morbidity and mortality. A recent clinical trial found that early rhythm control, mainly using anti-arrhythmic drugs (AADs), could benefit patients by lowering the risk of adverse cardiovascular events [1]. Aside from AADs, catheter ablation is a proven strategy for sinus rhythm (SR) maintenance [2]. Of the various catheter ablations, cryoballoon ablation (CBA) is one of the most well-established modalities for pulmonary vein isolation (PVI) to treat paroxysmal AF. CBA has comparable durability and safety to radiofrequency ablations [3]. Recent clinical trials have even reported the superiority of CBA over AADs when used as an initial therapy for preventing AF recurrence in patients with paroxysmal AF [4,5].

However, most randomized clinical trials on CBA have been conducted in Western countries [6,7]. Therefore, the efficacy and safety of CBA in maintaining SR in Asian populations remain unclear. A retrospective cohort study that enrolled paroxysmal AF patients undergoing CBA found that early recurrence of atrial tachyarrhythmias is the strongest predictor of AF recurrence, but the baseline parameters of AF recurrence were not analyzed [8]. Another retrospective study on Chinese AF patients suggested that a larger left atrium (LA) size and certain types of cryoballoon are related to long-term recurrence [9]. Similar studies on Asian populations remain limited. Whether the above predictors can be applied to Asian populations is unclear. Since the number of patients receiving CBA for AF is growing, identifying appropriate patients according to the recurrence predictors could help physicians to select a more suitable treatment strategy in practical terms, in particular, when deciding between AADs and CBA.

In the present study, we retrospectively analyzed consecutive patients with paroxysmal and persistent AF undergoing CBA, and then followed up these patients for one year. We aimed to determine the efficacy and the predictors of AF recurrence within this post-CBA time window in this Asian cohort. The results could assist physicians in choosing a suitable treatment strategy based on AADs or CBA for AF patients.

## 2. Materials and Methods

This study was conducted in accordance with the Declaration of Helsinki and approved by the Institutional Review Board of Taichung Veterans General Hospital (protocol code SE18171A) on 7 June 2018.

### 2.1. Study Population

We retrospectively analyzed 120 consecutive patients with paroxysmal or persistent AF undergoing CBA at our hospital from November 2014 to September 2020. Paroxysmal AF is defined as AF that terminates spontaneously or with intervention within 7 days of onset [10]. Persistent AF is defined as AF sustaining for >7 days, including episodes that are terminated by pharmacologic or electrical cardioversion after 7 days or longer [10].

### 2.2. Study Protocol

All AF patients undergoing CBA were consecutively included for a preliminary analysis of their pre-procedural baseline characteristics. Prior to CBA, echocardiography was conducted for all patients to assess the left atrial diameter (LAD), left ventricular ejection fraction (LVEF), and the presence of left ventricular hypertrophy (LVH, defined as an LV interventricular septum or posterior wall thickness >11 mm). Cardioversion was performed in patients with severe symptoms. All patients then received PVI by CBA. Linear ablation of the cavo-tricuspid isthmus (CTI) was performed when clinical typical atrial flutter (AFL) had been documented. After the index procedure, patients received AADs for the first 3 months. After that, AADs were discontinued in the absence of signs of AF recurrence or frequent atrial premature complex (APC). All patients were followed up for at least one year.

### 2.3. Electrophysiological and Cryoballoon Ablation Procedures

Electro-anatomical mapping (EAM) was guided by Ensite NavX/Velocity (Abbott, Inc., St. Paul, MN, USA). The CBA procedure was similar to those reported earlier [3]. In brief, the operators attempted PVI by advancing the cryoballoon toward each pulmonary vein (PV). Under fluoroscopic guidance using a contrast injection, adequate PV occlusion was achieved. The tissue was then cooled with a liquid refrigerant-filled balloon. For those PVs with visible PV potentials, PVI was performed until the PV potentials had disappeared, and the time-to-isolation (TTI) was finally recorded. Another 2 min boost was applied following each successful PVI. If an accessory PV (APV) was observed in the EAM and left atrial angiogram, cryoballoon ablation for APV isolation was carried out accordingly. If AF was sustained after PVI, synchronized cardioversion was applied to restore SR. Lastly, in the case of documented typical AFL prior to the procedure, linear ablation of CTI was performed during SR to achieve a bi-directional block at the CTI.

### 2.4. Patient Follow-Up and Evaluation of Late AF Recurrence

Seven follow-up visits were planned 0.5, 1, 2, 3, 6, 9, and 12 months after the procedure. A 12-lead ECG was routinely performed at each visit. At 3, 6, 9, and 12 months, 24-h Holter’s monitoring was also performed. Recurrence of AF was defined as the recurrence of any atrial tachyarrhythmia, including AF, AFL and atrial tachycardia, during this year-long period after CBA.

### 2.5. Statistical Analyses

Continuous variables were represented as the mean ± standard deviation. Categorical variables were represented as percentages. Comparisons of continuous variables were conducted using the Student’s t-test. Multivariable Cox proportional hazard regression models were used to identify factors contributing to AF occurrence. The event risk was expressed by the hazard ratio (HR) and a 95% confidence interval (CI). According to the Kaplan–Meier method, a log-rank test was used to estimate the freedom from AF throughout the year-long period in patients with different baseline parameters. Statistical analyses were conducted using IBM SPSS Statistics for Windows, version 24.0 (Armonk, NY, USA: IBM Corp.). Statistical significance was set at *p* < 0.05.

## 3. Results

### 3.1. Baseline Characteristics

A total of 120 patients with drug refractory symptomatic paroxysmal and persistent AF, who had undergone CBA, were analyzed. Their baseline characteristics are summarized in Table 1. The proportion of patients with paroxysmal AF was 72.5%, and the proportion of male patients was 70%. Their mean age was 61.9 ± 9.3 years, and the mean LAD was 4.38 ± 0.71 cm. Their AF duration was 48 ± 79 months, and their CHA2DS2-VASc score was 1.97 ± 1.58. The recurrence group had more persistent AF (*p* = 0.001), received pre-procedural cardioversion (*p* = 0.049), and had a larger LA size (*p* = 0.035) when compared with the non-recurrence group.

### 3.2. Cryoballoon Ablations

The procedural characteristics are listed in Table 2. The mean duration of each cryoapplication was 143.9 ± 10.7 s in each PV. CTI ablation was performed in 116 patients. Regarding the duration of cryoapplication, the percentage of patients receiving CTI ablation, the TTI in each PV, or the numbers of cryo-application per PV, there was no difference between the recurrence and the non-recurrence groups. However, the percentage of patients receiving electrical cardioversion was lower in the recurrence group (30%), compared with the non-recurrence group (48.4%) (*p =* 0.013).

### 3.3. Ablation Outcomes

After one year, 74.2% of the entire cohort (89 out of 120 patients) were free from AF after the 90-day blanking period post CBA. Similarly, for patients with persistent AF, 51.5% (17 out of 33 patients) did not experience AF recurrence in the first year. No patients died or had a stroke after CBA in this cohort.

### 3.4. Subgroup Analyses of AF Recurrence after CBA

**Figure 1:**
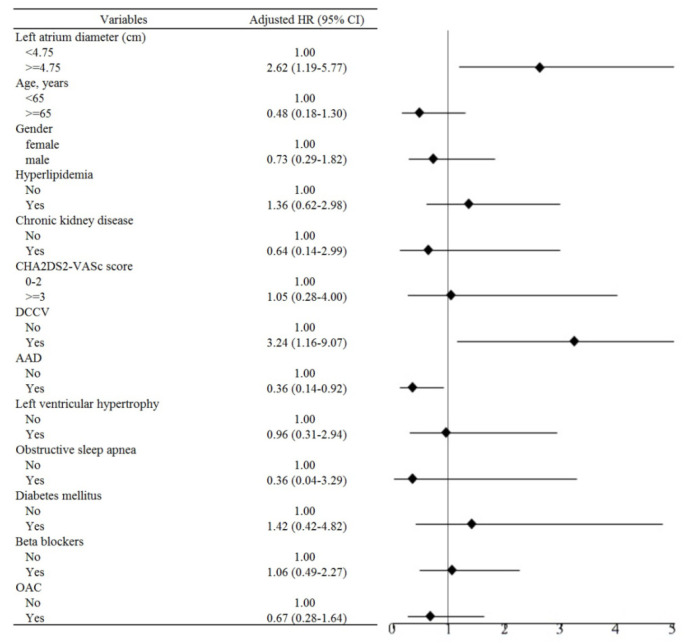
Subgroup analysis on the risk of AF recurrence after CBA. See text for abbreviations.

**Figure 2:**
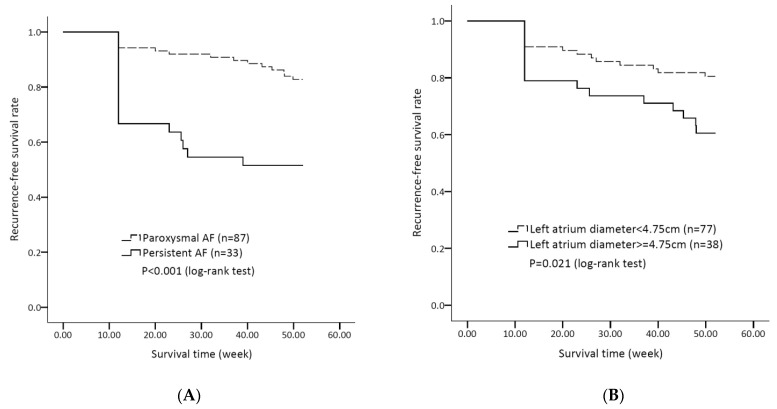
(**A**) Kaplan–Meier survival curves of AF recurrence after CBA in patients with paroxysmal or persistent AF in this cohort. (**B**) Kaplan–Meier survival curves of AF recurrence after CBA in patients with an LAD < 4.75 cm or LAD ≥ 4.75 cm. See text for abbreviations.

Table 3 shows the results of the subgroup analyses on patient characteristics and medications, regarding the risks of AF recurrence after CBA, based on a multivariate Cox hazards regression model. The risk of AF recurrence after CBA was higher in patients with persistent AF (adjusted HR 2.77, 95% CI 1.14–6.74, *p =* 0.025, model 2). Multivariate analyses revealed the association of three factors with AF recurrence: (1) the presence of pre-procedural cardioversion (adjusted HR 3.24, 95% CI 1.16–9.07, *p =* 0.025, model 3); (2) the use of AADs (adjusted HR 0.36, 95% CI 0.14-0.92, *p =* 0.032); and (3) an enlarged LAD ≥4.75 cm (adjusted HR 2.62, 95% CI 1.19–5.77, *p =* 0.016, model 3) (Figure 1). Figure 2A shows that patients with paroxysmal AF have a lower risk of AF recurrence after CBA, compared with those with persistent AF (log rank *p* < 0.001). Figure 2B shows that there are lower risks of AF recurrence after CBA in patients with an LAD <4.75 cm, when compared with those with an LAD ≥4.75 cm (log rank *p =* 0.021).

## 4. Discussion

The main findings of this study are as follows: (1) following CBA, 74.2% of Asian patients with paroxysmal and persistent AF did not experience AF recurrence after the blanking period; (2) no patients died or were diagnosed with a stroke after the procedure; (3) three factors, namely, the presence of persistent AF, an LAD ≥ 4.75 cm, and pre-procedural cardioversion, were associated with AF recurrence after CBA.

### 4.1. Efficacy of CBA for AF in Asian and Western Populations

CBA in paroxysmal AF patients has a treatment efficacy comparable to radiofrequency ablation in achieving PVI and SR maintenance [3]. Previous studies have reported an incidence from 25.2% to 31.3% of AF recurrence after CBA in Western populations [7,11,12]. In this Asian cohort, the incidence of AF recurrence after CBA was 25.8% after the blanking period. This percentage is consistent with the results from Western studies. Furthermore, there was no adverse event in our cohort, indicating that CBA in Asian populations is a feasible and safe strategy to maintain SR.

### 4.2. LA Size and AF Recurrence in Asian Populations

In this Asian cohort, we also determined the cutoff value of the LAD to predict AF recurrence after CBA. A recent Western single-center retrospective study on 542 AF patients found that an LAD >4.0 cm is a predictor of AF recurrence within one year [13]. Another meta-analysis on mostly Western populations, using CBA for AF, showed that an LAD >4.5 cm predicts AF recurrence [14]. In our present cohort, the patients had a more dilated LA (4.38 ± 0.71 cm) compared with the previous study, and 46.1% of our patients had an LAD >4.5 cm. Such a discrepancy indicates that the LAD >4.5 cm observed in Western populations might not be a good cutoff value for our patients. Based on the receiver operating characteristics, we, instead, observed that a cutoff LAD value of 4.75 cm predicts AF recurrence after CBA. Such a finding has not yet been reported in Asian cohorts. Our finding is also consistent with previous studies, in that a dilated LA is predictive of AF recurrence after CBA, with the only population-related difference being the exact cutoff value of the LA [6,7].

Several mechanisms have been proposed to explain the higher AF recurrence rate in AF patients receiving CBA. An enlarged LA might pose a technical challenge when fully occluding the PV–atrium junction, since the size of the balloon is fixed. AF patients with a large LA had more non-PV foci, which may not be eliminated by CBA [15]. A retrospective study in Japan also showed that CBA for AF caused a higher AF recurrence rate than radiofrequency ablation, particularly for AF patients with a dilated LA [16]. Further prospective studies are warranted to evaluate if LAD values ≥4.75 cm robustly predict AF recurrence after CBA in Asian populations.

### 4.3. CBA in Patients with Persistent AF

Persistent AF is characterized by an increased AF burden, longer AF duration, and larger extents of diseased substrate and atrial remodeling compared with paroxysmal AF. Persistent AF is, therefore, considered less susceptible to catheter ablation using PVI alone. Additional procedures, such as substrate modification, are usually required to achieve better results. To date, cryoballoon ablation has proven its non-inferiority to radiofrequency ablation for paroxysmal AF, based on the recurrence of atrial arrhythmia up to one year later [3]. In patients with persistent AF, similar data are less clear. The success rate of catheter ablation for persistent AF is reported to be 45–60% after one year [17]. In a prospective study on persistent AF, Straube et al. reported that 82% of their patients had not experienced atrial arrhythmia recurrence one year after CBA [18]. Another prospective trial by Su et al., using CBA on patients with persistent AF, found that 54.8% of them did not experience atrial arrhythmia recurrence [19]. Our present finding that 51.5% of patients (17 out of 33) are free of AF recurrence after one year is consistent with the results obtained by Su et al. Since the above three studies have a similar age distribution, LAD, LVEF, and clinical follow-up intervals, we speculated that such a discrepancy in the recurrence rate might be explained by the AF duration. Both our study and that of Su et al. have cohorts sharing a similar AF duration (5.1 and 6.4 years, respectively). In the study conducted by Straube et al., no information on AF duration was provided. In a meta-analysis, a trend was observed in that a shorter AF diagnosis to ablation time was associated with a lower rate of AF recurrence after catheter ablation [20]. The exact cause of this discrepancy in recurrence rates needs more studies to elucidate.

### 4.4. The Effect of Cardioversion on AF Recurrence

We found that pre-procedural cardioversion, implying severely bothersome symptoms, EHRA III, or IV, was another independent factor for predicting AF recurrence after CBA. Specifically, the persistent AF group (6 out of 33, 18.2%) received more cardioversion than its counterpart group (3 out of 87, 3.4%) (*p =* 0.013). The impact of pre-procedural cardioversion may be explained by its greater effect on patients with persistent AF. As cardioversion and persistent AF were independent predictors in this study, these results suggest that these symptoms are also likely to contribute to AF recurrence after CBA. On the other hand, some patients were highly symptomatic, but had experienced a relatively short duration between AF diagnosis and ablation; therefore, they fell into the category of persistent AF. In these patients, cardioversion still played a critical role in alleviating the patients’ discomfort. The severity of symptoms, as part of the “4S scheme,” has not been shown to be associated with AF recurrence after catheter ablation [10]. Our findings suggest that more data are needed to clarify this relationship.

### 4.5. Strengths and Limitations

Our study has some limitations. Firstly, this is a single-center retrospective analysis with a limited number of patients and a short follow-up duration. However, our physicians followed the same CBA protocol throughout the study period to ensure consistency. Secondly, Holter’s ECG monitoring was arranged periodically in our clinic. However, we could not exclude the possibility that patients might experience a recurrence of paroxysmal asymptomatic AF. Thirdly, this study was conducted in an Asian population. Although the results are consistent with those obtained in studies on Western populations, extending our findings to other ethnic populations should be performed with caution. Fourthly, the use of AADs was at the discretion of physicians, and could be confounded by symptom severity, underlying comorbidities, physicians’ predilection and patients’ preferences. Finally, the number of patients with persistent AF is small, and applying this study result to patients with persistent AF should also be performed with caution.

## 5. Conclusions

Based on our single-center study, with a relatively small sample size, we found that CBA is an effective and safe procedure in Asian patients with AF. The presence of persistent AF, a large LA size (≥4.75 cm), and severe symptoms are three predictors of AF recurrence after CBA after one year. A cutoff value of 4.75 cm for the LAD might identify suitable AF patients for CBA ablation in Asian populations.

## Figures and Tables

**Table 1:** Baseline characteristics in patients with and without AF recurrence.

	Total (*N* = 120)	Non-Recurrence (*N* = 89)	Recurrence (*N* = 31)	*p*
Baseline demographics				
Male	84 (70.0%)	66 (74.2%)	18 (58.1%)	0.092
Age (y)	61.9 ± 9.3	62.3 ± 9.0	61.0 ± 10.2	0.504
Age ≥ 65 years	52 (43.3%)	41 (46.1%)	11 (35.5%)	0.306
Body mass index	25.98 ± 3.76	25.9 ± 3.6	26.3 ± 4.2	0.529
Left ventricular ejection fraction (%)	55.9 ± 8.5	56 ± 8.9	55.4 ± 7.5	0.746
Left atrium diameter (cm)	4.38 ± 0.71	4.4 ± 0.7	4.7 ± 0.8	0.035
Left atrium enlargement ≥ 45 mm	53 (46.1%)	36 (42.4%)	17 (56.7%)	0.176
NYHA functional class	0.975 ± 0.399	1.0± 0.4	1.0± 0.4	0.355
AF characteristics				
AF duration (months)	48.0 ± 78.9	44.9 ± 70.7	57.7 ± 101	0.546
AF duration ≥3 years	41 (37.3%)	30 (36.1%)	11 (40.7%)	0.668
Paroxysmal AF	87 (72.5%)	72 (80.9%)	15 (48.4%)	0.001
Persistent AF	33 (27.5%)	17 (19.1%)	16 (51.6%)	0.001
Prior electrical cardioversion	9 (7.5%)	4 (4.5%)	5 (16.1%)	0.049
Comorbidities				
Left ventricular hypertrophy	100 (86.2%)	74 (86%)	26 (86.7%)	1.000
Valvular heart disease	67 (57.8%)	47 (54.7%)	20 (66.7%)	0.251
Obstructive sleep apnea	4 (3.3%)	3 (3.4%)	1 (3.2%)	1.000
COPD	0 (0%)	0 (0%)	0 (0%)	-
Hypertension	66 (55%)	47 (52.8%)	19 (61.3%)	0.414
Hyperlipidemia	48 (40%)	34 (38.2%)	14 (45.2%)	0.496
Diabetes mellitus	15 (12.5%)	11 (12.4%)	4 (12.9%)	1.000
Chronic kidney disease	11 (9.2%)	9 (10.1%)	2 (6.5%)	0.727
Stroke or TIA	17 (14.2%)	14 (15.7%)	3 (9.7%)	0.555
CAD	29 (24%)	25 (28.1%)	4 (12.9%)	0.089
CABG	1 (0.8%)	1 (1.1%)	0 (0%)	1.000
CHA2DS2-VASc score	1.97 ± 1.58	2± 1.6	2 ± 1.6	0.997
Medications				
Anti-platelet agents	41 (34.2%)	29 (32.6%)	12 (38.7%)	0.536
CCB	32 (26.7%)	20 (22.5%)	12 (38.7%)	0.078
Beta blockers	53 (44.2%)	39 (43.8%)	14 (45.2%)	0.897
ACEi/ARB	42 (35%)	30 (33.7%)	12 (38.7%)	0.615
OAC	79 (65.8%)	59 (66.3%)	20 (64.5%)	0.858
AADs	101 (84.2%)	78 (87.6%)	23 (74.2%)	0.091
Class I AADs	56 (46.7%)	46 (51.7%)	10 (32.3%)	0.094
Class III AADs	51 (42.5%)	36 (40.4%)	15 (48.4%)	0.528

Abbreviations: AF, atrial fibrillation; NYHA, New York Heart Association; COPD, chronic obstructive pulmonary disease; TIA, transient ischemic attack; CAD, coronary artery disease; CABG, coronary artery bypass surgery; CCB, calcium channel blocker; ACEi, angiotensin-converting enzyme inhibitor; ARB, angiotensin II receptor blocker; OAC, oral anticoagulant; AADs, anti-arrhythmic drugs.

**Table 2:** Cryoablation parameters in patients with and without recurrence (*N* = 120).

	Total	Non-Recurrence (*N* = 89)	Recurrence (*N* = 31)	*p* *
Duration of each cryoapplication (s)	143.9 ± 10.7	144.0 ± 11.4	143.8 ± 8.3	0.95
Cavo-tricuspid linear ablation	116 (96.7%)	87	29	0.27
Time-to-isolation (s)				
LSPV (*n* = 92)	52.9 ± 28.5	53.7 ± 25.9	50.7 ± 35.9	0.67
LIPV (*n* = 76)	36.5 ± 20.9	36.0 ± 21.6	37.8 ± 19.0	0.74
RSPV (*n* = 76)	32.6 ± 24.1	33.0 ± 24.8	30.9 ± 22.1	0.75
RIPV (*n* = 38)	36.9 ± 24.9	37.3 ± 26.4	35.9 ± 22.6	0.88
Number of cryoapplications per vein	2.1 ± 0.3	2.1 ± 0.4	2.1 ± 0.2	0.98
Electrical cardioversion during the cryoablation procedure	36 (30%)	21 (23.6%)	15 (48.4%)	0.013
Safety endpoints				
Death	0 (0%)	0	0	–
Stroke or TIA	0 (0%)	0	0	–
Phrenic nerve injury	2 (1.7%)	1	1	–
Cardiac tamponade requiring pericardiocentesis	2 (1.7%)	1	1	–

* Non-recurrence group versus recurrence group; abbreviations: LSPV, left superior pulmonary vein; LIPV, left inferior pulmonary vein; RSPV, right superior pulmonary vein; RIPV, right inferior pulmonary vein; TIA, transient ischemic attack.

**Table 3:** The effect of baseline characteristics and medications on the risk of AF recurrence after CBA.

Variables	Event	PYs	Rate	Model 1	*p*	Model 2	*p*	Model 3	*p*
AF									
Paroxysmal AF	15	4190.2	3.6	Ref	-	Ref	-	-	-
Persistent AF	16	1156.6	13.8	3.52 (1.73–7.15)	0.001	2.77 (1.14–6.74)	0.025	-	-
LAD (cm)									
<4.75	15	3564.86	4.2	Ref	-	-	-	Ref	-
≥4.75	15	1561.94	9.6	2.21 (1.08–4.52)	0.030	-	-	2.62 (1.19–5.77)	0.016
LAD (cm)									
<4.5	13	2836.86	4.6	Ref	-		-	-	-
≥4.5	17	2289.94	7.4	1.59 (0.77–3.28)	0.205	-	-	-	-
Age, years									
<65	20	2903.3	6.9	Ref	-	Ref	-	Ref	-
≥65	11	2443.5	4.5	0.67(0.32–1.40)	0.291	0.62 (0.22–1.74)	0.368	0.48 (0.18–1.30)	0.150
Gender									
female	13	1564.8	8.3	Ref	-	Ref	-	Ref	-
male	18	3782	4.8	0.58(0.28–1.18)	0.132	0.70 (0.28–1.72)	0.435	0.73 (0.29–1.82)	0.495
Hyperlipidemia									
No	17	3207.2	5.3	Ref	-	Ref	-	Ref	-
Yes	14	2139.6	6.5	1.24(0.61–2.52)	0.551	1.13 (0.52–2.46)	0.760	1.36 (0.62–2.98)	0.437
CKD									
No	29	4854.8	6	Ref	-	Ref	-	Ref	-
Yes	2	492	4.1	0.68(0.16–2.84)	0.596	0.62 (0.13–2.86)	0.538	0.64 (0.14–2.99)	0.571
CHA2DS2-VASc									
0–2	20	3733.3	5.4	Ref	-	Ref	-	Ref	-
≥3	11	1613.5	6.8	1.28(0.61–2.66)	0.518	1.52 (0.43–5.40)	0.521	1.05 (0.28–4.00)	0.942
Cardioversion									
No	26	5049.8	5.1	Ref	-	Ref	-	Ref	-
Yes	5	297	16.8	2.92(1.12–7.63)	0.029	2.58 (0.90–7.44)	0.079	3.24 (1.16–9.07)	0.025
AADs									
No	8	739.74	10.8	Ref	-	Ref	-	Ref	-
Yes	23	4607.06	5	0.48(0.22–1.08)	0.078	0.69 (0.25–1.93)	0.481	0.36 (0.14–0.92)	0.032
LVH									
No	4	697	5.7	Ref	-	Ref	-	Ref	-
Yes	26	4481.8	5.8	1.02(0.36–2.92)	0.972	1.29 (0.40–4.10)	0.668	0.96 (0.31–2.94)	0.942
OSA									
No	30	5170.8	5.8	Ref	-	Ref	-	Ref	-
Yes	1	176	5.7	0.99(0.14–7.28)	0.994	0.56 (0.06–4.81)	0.596	0.36 (0.04–3.29)	0.365
DM									
No	27	4664.34	5.8	Ref	-	Ref	-	Ref	-
Yes	4	682.46	5.9	1.02(0.36–2.92)	0.968	0.95 (0.27–3.29)	0.932	1.42 (0.42–4.82)	0.577
Beta blockers									
No	17	3018.94	5.6	Ref	-	Ref	-	Ref	-
Yes	14	2327.86	6	1.06(0.52–2.15)	0.873	0.94 (0.44–2.04)	0.885	1.06 (0.49–2.27)	0.887
OAC									
No	11	1793.16	6.1	Ref	-	Ref	-	Ref	-
Yes	20	3553.64	5.6	0.93(0.44–1.93)	0.837	0.62 (0.26–1.49)	0.285	0.67 (0.28–1.64)	0.381

Model 1: crude HR. Model 2 and Model 3: adjusted HR for age, gender, hyperlipidemia, CKD, CHA2DS2-VASc score, DCCV, AADs, LVH, OSA, DM, beta blockers, and OAC use. PYs: person-years, per 1000 PYs. Abbreviations: AF, atrial fibrillation; CBA, cryoballoon ablation; LAD, left atrium diameter; CKD, chronic kidney disease; AADs, anti-arrhythmic drugs; LVH, left ventricular hypertrophy; OSA, obstructive sleep apnea; DM, diabetes mellitus; OAC, oral anticoagulant.

## Data Availability

All relevant data are presented within the paper.
